# Can longer gaze duration determine risky investment decisions? An interactive perspective

**DOI:** 10.16910/jemr.14.4.3

**Published:** 2021-09-21

**Authors:** Yiheng Wang, Yanping Liu

**Affiliations:** Institute of Applied Psychology, Guangdong University Finance, China; Department of Psychology, Sun Yat-sen University, China

**Keywords:** Eye movement, eye tracking, risky decision, dual-contribution model, attention, gaze cascade effect

## Abstract

Can longer gaze duration determine risky investment decisions? Recent studies have tested how gaze influences people's decisions and the boundary of the gaze effect. The current experiment used adaptive gaze-contingent manipulation by adding a self-determined option to test whether longer gaze duration can determine risky investment decisions. The results showed that both the expected value of each option and the gaze duration influenced people's decisions. This result was consistent with the attentional diffusion model (aDDM) proposed by Krajbich et al. (2010), which suggests that gaze can influence the choice process by amplify the value of the choice. Therefore, the gaze duration would influence the decision when people do not have clear preference.The result also showed that the similarity between options and the computational difficulty would also influence the gaze effect. This result was inconsistent with prior research that used option similarities to represent difficulty, suggesting that both similarity between options and computational difficulty induce different underlying mechanisms of decision difficulty.

## Introduction

Decisions are made almost every second. For example, we must decide which goods to purchase, which direction to explore, and which risks to avoid. Many studies have examined the decision-making process, and many models predict people’s decisions under various conditions (6 ; 25). However, the degree to which the bottom-up process can influence these decisions remains unknown, especially when the decisions involve uncertainty and risk in investment activities (23). The current study is aim to test the boundary of the gaze-orientation effect on risky investment decisions.

Risky decisions are used to study how people make decisions, and many models have been provided. For example, the expected utility model suggests that people are rational and make decisions by evenly calculating and comparing the value of each option to receive maximal benefits (25). Later research indicated that people use heuristic processes to make decisions as per the noncompensatory model (19, 20). Heuristics as shortcuts can reduce the complexity of decision making by allowing the decision maker to focus on the most critical information (4). However, more recent studies have found evidence that contradicts the holistic model (3; 5; 8 ; 9; 11). Recent study done by Su et al., (22) compare the calculation process with the risk choice process, and their result showed that the eye-movement of risky choice task were different from the mere calculation process. These results suggested that many factors could influence people’s decisions. For example, people may be distracted by salient stimuli (salience effect), surface characteristics (framing effect), and subconscious processes (priming effect) (12; 13).

The latest studies have focused on the importance of gaze duration. A psychological phenomenon known as the mere-exposure effect (26 ), suggests that extending exposure can induce individual preferences. Based on this phenomenon, Shimojo et al. (17) presented the gaze-cascade model, showing a positive relationship between fixation duration and preference for a particular face, suggesting that people’s fixation duration can influence their preferences. Many studies showed that the longer fixation can predicted people’s preference on pictures (16; 10). Researcher further manipulated the presented time of each option to test the causal relationship between the fixation and people’s preference on face (17); foods (1); and products (13).

However, previous studies testing the causal relationship between gaze duration and final choice were limited in that they only manipulated the presentation time of each stimulus. This enables participants to detect the intent of these studies, which may cause demand effects (14). Therefore, to effectively avoid such demand effects, Pärnamets et al. (15) developed a novel gaze-contingent prompt paradigm, which passively manipulates participants’ gaze times. Using the eye-tracking technique to record participants’ eye movements, participants were presented two alternative options simultaneously and were required to make a decision when their gaze on the randomly selected option (target option) reached the time limit. This paradigm assumes that people will accumulate stochastic evidence for one of the two options and thus make their decision when the accumulated evidence reaches a threshold. Therefore, the option that includes a longer process would become more preferable. Many researchers have used this paradigm, showing that gaze duration can manipulate moral judgment decisions (15), perceptual judgments (14), gambles (21), and risky choices (2; 23).

However, research has also suggested that the effect of gaze duration has a boundary. For example, Shimojo et al. (17) found that the gaze cascade effect was stronger when participants were asked to choose their preference between two faces with similar attractiveness than to decide which face was rounder. Another study found that the cascade effect was stronger for neutral stimuli than for more extreme values (1 ). Newell & Le Pelley (14) showed that the bias effect existed only among impossible trials and not among possible trials. In their experiment, participants were asked to determine which picture contained more dots. For the impossible trials, participants were shown two pictures that each contained a very similar number of dots (e.g., 101 vs 102 dots). For the possible trials, participants were shown two pictures that each had a very different number of dots (e.g., 1 vs 100 dots).

These different results for perceptual and moral tasks indicated that both top-down and bottom-up processes can simultaneously influence decisions as stipulated by the dual-contribution model (17 ). This model assumes that both the cognitive assessment system and orienting behaviors influence decisions, while gaze information mainly influences decisions through the bottom-up process. The attentional diffusion model (aDDM) proposed by Krajbich et al. (2010) further suggested that the bottom-up process (i.e. the gaze attention) guide the top-down process (i.e.the choice value) by amplify the choice value (18). Many studies supported the idea and showed that there were some effects of bottom-up information (e.g., gaze position and duration) biasing the top-down process (e.g., attention) on decision making (e.g. 24). In addition, Ghaffari and Fiedler (7) adapted the gaze-contingent prompt paradigm by allowing participants to choose an option before the prompt (self-determined choice). Their result showed that the self-determined trials were appear when participants decided not to choice the target option and when participants were confident about their decision. These studies show that decision making may involve interactive processes.

Following this logic, gaze information may influence decisions that are difficult for people to make (or lack a top-down process). However, previous research primarily focused on studying the gaze bias effect on more subjective decision-making tasks and only used similarity between options as an indicator of decision difficulty. To date, relatively little is known about how interactive processes influence risk-investing decisions, especially when the task is computationally demanding. The latest study demonstrated only that gaze duration could determine risky decisions as other tasks (23). The investment tasks required making a complex decision by simultaneously considering both the absolute reward and its uncertainty. This enables comparing the top-down and bottom-up processes in the decision making process. In this way, the current study adapted the gaze-contingent prompt paradigm by adding a self-determined option to further examine the effect of gaze on risky investment decisions after controlling the top-down process. Finally, risky investment decisions allow exploring the effect of gaze duration with a different difficulty indicator: computational difficulty. The complex computation process may increase the difficulty in making decisions, which will enable exploring the boundary of the gaze-cascade effect.

## Methods

### Participants and apparatus

Describe who participated in your study. How many participants were in the study and how were they selected/recruited? In what way were the participants compensated for running in the study? Were any data sets deleted? If so, why were they deleted? Describe any demographics of the participants that important to the study. If you’ve conducted an experiment, indicate how many participants were assigned to each condition.

Based on prior effect size (= 0.6) as reported by Newell and Le Pelley (14) and Pärnamets et al. (15), the G*power (Faul et al., 2007) results showed that the t-test comparing difference from constant could reach 90% power with 39 participants. Three participants did not finish the task and were excluded from the final analysis. In all, forty-two participants (13 men, mean age = 20.14 years) were recruited from the subject pool of Sun Yat-sen University.

All participants were required to sit in front of a 27-inch screen at a 70-cm distance (resolution = 2560 × 1440 pixels, refresh rate = 144 Hz). Stimuli were presented by the OpenGL-based psychophysics toolbox 3 and the EyeLink toolbox extension based on MATLAB. The EyeLink 1000 eye tracker was used to record participants’ right eye movement while the view was binocular. Five-point calibration and validation were performed before starting the experiment, with the maximal calibration and validation error less than 0.4° of the visual angle.

### Design and materials

**Figure 1.**
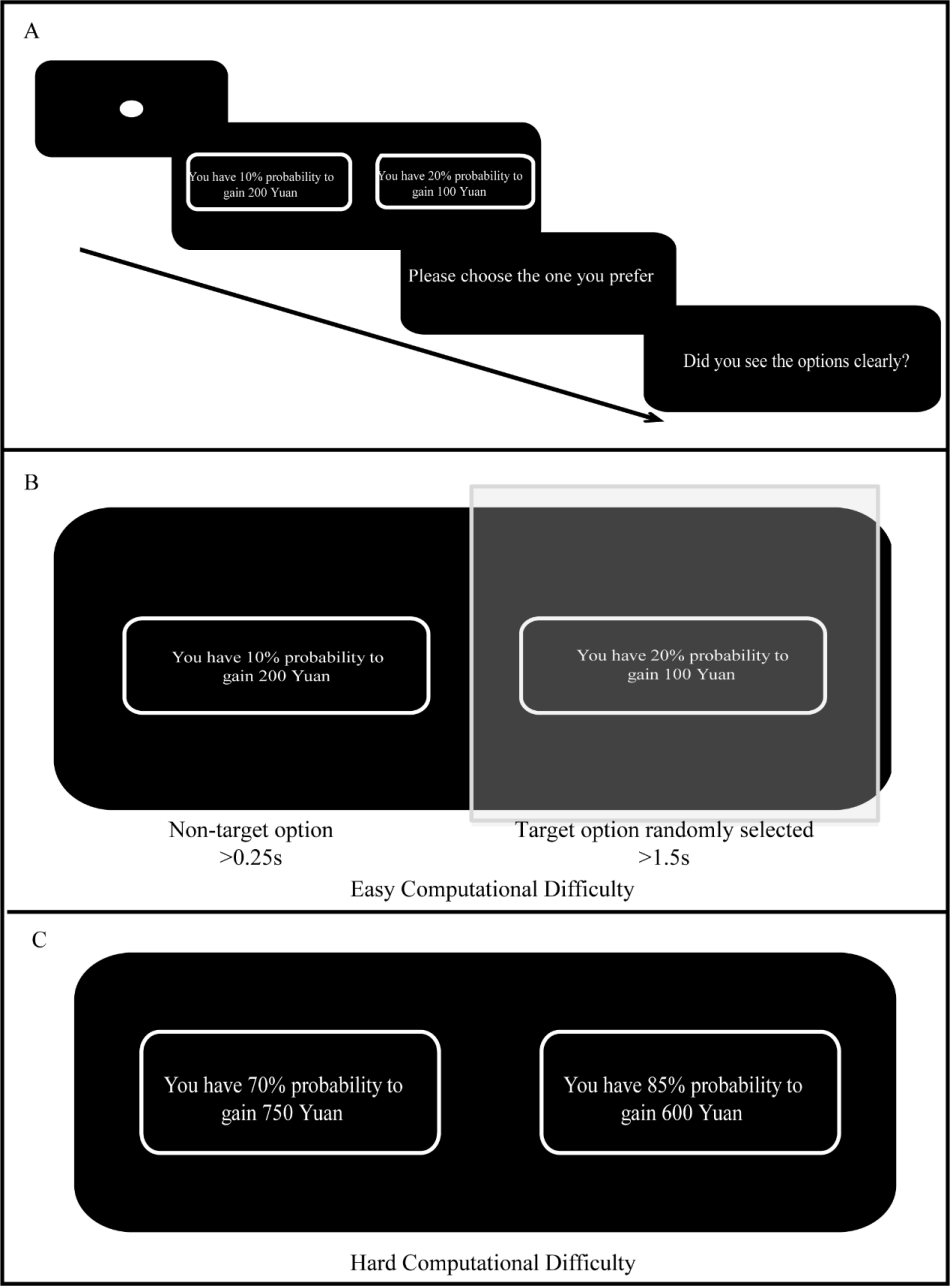
Experimental procedure (A) and trigger condition (B), and stimuli example for each condition (C). The question prompted when the target option determined randomly before each trial was viewed at least 1.5s and the non-target option was viewed at least 0.25s in the experiment. Note. The sample as presented was translated from Chinese to English just for an illustrative proposal.

Participants chose one of the alternative options with different proportions and different monetary amounts (Figure 1). Two options were presented simultaneously and horizontally on both sides of the screen, with 160 pixels between them. As suggested by Newell and Le Pelley (14), various task difficulties needed to be considered in the gaze-contingent bias experiments; thus, we manipulated the task difficulty with easy/hard levels. Specifically, the hard computational condition contained 48 hard trials, and the contrast condition contained 48 easy trials.For the hard condition, the option was consisted of value was single-digit number multiplied by 100 with probability was two-digit number multiplied by 10(e.g.15% of 100); or the value was two-digit number multiplied by 100 with the probability was single-digit number multiplied by 10(e.g.10% of 150). For the easy condition, the option was consisted of value was single-digit number multiplied by 100 with probability was also single-digit number multiplied by 10 (e.g. 10% of 200). Prior research (17) indicated that the gaze effect would be stronger when both options were similar. Therefore, we recorded the possibility of each option, which was defined as the expected value difference between the two options (the higher possibility the lower similarity). Each pair of stimuli was repeated twice for a total of 192 trials. Participants voluntarily participated in the 20-30 minute experiment in exchange for 15–30 yuan (M=23.20, SD=3.97) (approximately $2 to $4). The payment was calculated by summing up the total money they get in each trial and then times 0.01. For each trial, participants can get the money if a random number generated by MATLAB was larger than the probability they choose. The detail instruction can be found in the supplementary materials.

### Procedure

After the calibration and validation procedure, participants were instructed to decide between two alternative options by using a joystick (Figure 1). For each trial, participants were presented two options on the screen while the eye tracker began recording.The current study adapted the gaze-contingent paradigm that the decision was prompt when participants’ fixation on each options were reach the absolute duration, they stated that people need 0.25 to clearly see two options while 3s is needed to make decisions. The question page (“Please indicate the option you prefer.”) would prompt when the target option had been viewed for at least 1.5 s, or the non-target option had been viewed for at least 0.25 s, or when the trial had lasted for 3 s. A pilot was conducted to test the parameters. The pilot test showed that for 750 trials, the minimum time spend on a decision trial were 0.25s, the average time spend on a decision trial were 3.03s. And after 15 practice trials, the average time spend on the selected option was 1.5s. Unlike the gaze-contingent paradigm, participants can used the joystick to select one of the two alternatives once they had decided (self- determined trials) in the current study. After each choice, participants were asked whether they had seen the options clearly by using a 5-point scale (1 = not clear at all; 5 = totally clear). Participants had five trials to practice the procedure and could take a short break after every 48 trials.

## Results

Trials were excluded if participants reported that they could not see the options (clarity = 1). The self-determined rate was 22.4%, and the timeout rate was 1.4% for 8004 trials total. As suggested by Newell & Le Pelley (14), the timeout trials were included in the analysis. Therefore, the current experiment analyzed the proportion of target options chosen under the different conditions when the self-determined trials were included and excluded. Mixed-effect models with the random effects of participants and stimuli were conducted to test the gaze-cascade effect on investment decision making.

### Success of the gaze-cascade effect

Prior research showed that passively manipulating participants’ gaze duration allowed them to fixate on non-target options longer than on the target option. We defined successfully manipulated trials as those with a longer gaze duration on the target options than on the non-target options. The success rate for the manipulated trials was 55.3% for all trials. When the self-determined trials were excluded, the success rate increased to 60.3%.

### Proportion of choosing the target option

When self-determined trials were included, the one sample t-test indicated that the proportion of trials in which the target option was chosen (*M*=51.10%, *SD*=3.53) was significantly higher than that if the choice had been random (50%, *p = .*050). When self-determined trials were excluded, the proportion of trials in which the target option was chosen (*M* = 54.07%, *SD* = 8.29) was significantly higher than that if the choice had been random (50%, *p =* .003). For the self-determined trials, the proportion of trials in which target option was chosen (*M* = 35.71%, *SD* = 22.36) was significantly lower than that if the choice had been random (50%, *p =* .001).

### Proportion of choosing the target option under different conditions

When self-determined was included, the one sample t-test indicated that the proportion of trials in which the target option was chosen (*M* = 52.35%, *SD* = 4.90) was significantly higher than that if the choice had been random (50%, *p = .*003) for the easy condition. While the proportion did not differ for the hard condition (*M* = 49.85%, *SD* = 8.29, *p = .*866). In addition, the one sample t-test indicated that the proportion of trials in which the target option was chosen(*M* = 52.53%, *SD* = 3.85) was significantly higher than that if the choice had been random (50%, *p < .*001) for the low possibility condition(i.e. expected value difference smaller than 15), while the proportion did not differ for the high possibility condition (*M* = 50.04%, *SD* = 4.86, *p = .*955).

When self-determined trials were excluded, the proportion of trials in which the target option was chosen (*M* = 55.69%, *SD* = 9.05) was significantly higher than that if the choice had been random (50%, *p < .*001) for the easy condition, but this proportion did not differ for the hard condition (*M* = 52.33%, *SD* =9.35, *p = .*113). The proportion of trials in which the target option was chosen was significantly higher than that if the choice had been random (50%) in both high (*M* = 53.06%, *SD* = 9.30) and low possibility condition (*M* = 55.69%, *SD* = 8.79), but the effect was stronger among the low possibility condition (*p < .*001), as compare to the high possibility condition (*p* = .039).

### Effects of the top-down and bottom-up processes

Mixed-effect models using the lme4 package (Bates et al., 2015) were conducted to compare the top-down (higher expected value) and bottom-up (target position) processes during the investment decision-making tasks. The generalized linear mix effect model was conducted to examine the participants’ final choice (A = 0; B = 1) was predicted by the target position (A = 0; B = 1), the advance expected value of option B, and their interaction. For the random effect, the stimuli and participants were fit into the random intercept, while the advance expected value was fitted into the random slope varying across participants (see table 1). The result showed that the interaction effect was not significant, but the target position (*b* = 0.16, *SE* = 0.06, *p* = .004), and the advance expected value of option B (*b* = 1.76, *SE* = 0.18, *p* < .001) can both significantly predicted the final choice.

**Table 1.** Generalize linear mixed model predicting final choice.

Fixed effects:	Estimate	SE	z	p
(Intercept)	-0.21	0.13	-1.65	0.1
Target	0.16	0.06	2.84	0.004
Advance Expected value of B option	1.76	0.18	10.01	<.001
Target x Advance Expected value of B option	-0.03	0.09	-0.32	0.753
Random effects:	Variance	SD	Cor.	
(Intercepts)	Stimulus	2.35	1.53	
	Participants	0.11	0.33	
Slopes (varying over participants)	Advance Expected value	0.46	0.68	0.24

Note. R model equation: Choice~Target*AdvEV-B+(1|StimuliN)+(1+AdvEV|IDN)

In addition, the generalized linear mix effect model was conducted to examine whether the task difficulty, possibility,the expected value of the target option, the timeout and self-determine trials would influence the gaze-cascade effect. The stimulus and participants were fitted into the model as random intercept (see table 2). The result showed that people would tend to select the target option when the target options are easy (*b* = -2.53, *SE* = 0.63, *p* < .001), with similar expected value difference (*b* = -0.07, *SE* = 0.01, *p* < .001) and have higher expected value (*b* = 5.76, *SE* = 0.27, *p* < .001). The result also showed that the gaze-cascade effect was stronger when the trials are not self-determined (*b* = -0.30, *SE* = 0.07, *p* < .001) and not time-out (*b* = -0.77, *SE* = 0.22, *p* < .001).

**Table 2.** Generalized linear mixed model predicting choice the target option

Fixed effects	Estimate	SE	z	p
(Intercept)	6.31	1.02	6.16	<.001
Difficulty	-2.53	0.63	-4.02	<.001
Possibility	-0.07	0.01	-9.42	<.001
Advance Expected value of Target option	5.76	0.27	21.48	<.001
Self Determine	-0.30	0.07	-4.53	<.001
Timeout	-0.77	0.22	-3.5	<.001
Random effects:		Variance	SD	
(Intercepts)	Stimulus	18.19	4.26	
	Participants	0.004	0.06	

Note. R model equation: MatchTC~Difficulty+Possibility+AdvEV-T+Self-Determine+Timeout+(1|StimuliN)+(1|IDN)

## Discussion

This experiment manipulated task computational difficulty and examined whether the length of time spent looking at an option influenced investment decisions. The study design was the same as that used in previous studies except that this study allowed participants to self-determine when they had already decided. This adaptation increased the manipulation success rate. First, gaze manipulation influenced risky decisions when self-determined trials were both included and excluded. The effect was stronger when self-determined trials were excluded. Moreover, the target option was more likely to be chosen than was the non-target option in both difficulty (easy v.s hard) and possibility (small v.s large expected value difference) condition. Although the effect was stronger in the easy and small expected value difference condition as compare to the hard condition and large expected value difference condition. Finally, the mix effect logistic regression analysis showed that, the expected value and gaze manipulation can both influence participants final choice. Another mix effect logistic regression analysis showed that the gaze-cascade effect is stronger among the easy, smaller expected value difference and non-self-determine trials.

Sui et al. (23) found that gaze duration for the target option was shorter than that for the non-target option when using gaze-contingent manipulation; therefore, they suggest that the effectiveness of this paradigm should be improved. The current study adapted gaze-contingent manipulation by adding a self-determined option before the prompt as did Ghaffari and Fiedler (7). These authors used this setting to separate the top-down and bottom-up processes during decision making. However, they did not test whether this adaptation improved the effectiveness of the paradigm. The current study showed that adding the self-determined option increased the success rate of gaze-contingent manipulation. The timeout rate were also decrease in the current study as compare to the prior studies (e.g. 23; 14). Therefore, the current study found that this adaptation improved the effectiveness of the gaze-contingent paradigm.

Several researchers have debated whether gaze duration manipulates decision making. Some researchers suggest that gaze duration as a bottom-up process could influence decisions (15); other researchers suggest that gaze duration only reflects the top-down process of decision making (14). The current study revealed both cognitive assessment and orienting behaviors during decision making for risky investment decisions. Similar result was found among Ghaffari and Fiedler (7)’s study, suggesting that although bottom-up information exerts some effects, the gaze manipulation can only influence people’s decision when they had no preference. In addition, the current study showed that the gaze-cascade effect was stronger when the target choice have higher value. These results are consistent with the attentional diffusion model(aDDM) proposed by Krajbich et al. (2010), which suggests that gaze can influence the choice process by amplify the value of the choice (18)

The current study also found some surprising results. Prior research showed that the gaze-cascade effect was stronger when people had difficulty making decisions. The current study also showed that the gaze-cascade effect was stronger when the options are similar (i.e.small expected value difference). However, the current results also showed that the gaze-cascade effect was stronger under easy computational difficulty than under hard computational difficulty. Su et al. (22) found similar results, showing that when computational difficulty increases, people tend to rely more on weighing and adding processes, that is, to calculate the expected value of each option. Therefore,people’s attention might focus more on the calculation process among the hard computation task and less likely to be influenced by the gaze orientation. Further study is needed to test this possibility.

In summary, the current study is aim to test the boundary of the gaze-cascade effect on risky investment decisions. The results showed that after controlling the top-down process, the target option with a longer gaze duration was more likely to be chosen. Therefore, the gaze-cascade effect might be only effective when people do not have clear preference. In addition, the current study showed that the gaze-cascade effect was also limited among the hard computational difficulty tasks. It is possible that the hard computational tasks would attract people’s attention to the calculation process instead of the risky taking process. Future work should investigate the underlying mechanism of the gaze-cascade effect for different levels of decision difficulty induced by option similarity and computational difficulty.

### Ethics and Conflict of Interest

The author(s) declare(s) that the contents of the article are in agreement with the ethics described in http://biblio.unibe.ch/portale/elibrary/BOP/jemr/ethics.html and that there is no conflict of interest regarding the publication of this paper.

### Acknowledgements

This research was supported by the grants from the MOE (Ministry of Education in China) Project of Humanities and Social Sciences (18YJC190014), the Fundamental Research Funds for the Central Universities (19wkzd21) and Innovation Team Project of Guangdong Province Universities(2019WCXTD005).
